# WHAT IS AGEISM?: A REVIEW AND ANALYTICAL CRITIQUE OF CURRENT MEASURES OF AGEISM

**DOI:** 10.1093/geroni/igz038.3336

**Published:** 2019-11-08

**Authors:** Jerin Lee, Jenna Wilson, Natalie Shook

**Affiliations:** West Virginia University, Morgantown, West Virginia, United States

## Abstract

The past two decades have been marked by a rapidly aging population in the U.S. (U.S. Census Bureau, 2018), making prejudicial attitudes toward older adults (i.e., ageism) and the impact of such attitudes more relevant. Understanding ageism is necessary to change institutionalized beliefs and reduce prejudice toward older adults. However, it requires the availability of valid and reliable measures of ageism. The purpose of the present research was to: (1) provide an analytical review of three existing self-report measures of ageism (i.e., Fabroni Scale of Ageism [FSA]; Relating to Older People Evaluation [ROPE]; Ambivalent Ageism Scale [AAS]); and (2) examine the reliability and convergent validity of these ageism measures. A total of 473 undergraduate students completed the FSA, ROPE, and AAS online. The results indicated that the FSA, subscales of the ROPE (i.e., positive and negative ageism), and subscales of the AAS (i.e., benevolent and hostile ageism) were generally positively associated with one another, with two exceptions. First, positive ageism was negatively correlated with the FSA. Second, positive ageism was not significantly correlated with hostile ageism. Importantly, there was notable variability in the magnitude of the correlations between the measures, as correlations were mostly weak to moderate in magnitude (rs ranged from -.13 to .65). These associations are below the recommended threshold of r = ±.70 for convergent validity (Carlson & Herdman, 2012), suggesting conceptual problems with current ageism measures as they do not appear to reflect a common construct, which has practical implications for future theoretical and empirical work.

